# Evaluating risk factors for endemic human *Salmonella* Enteritidis infections with different phage types in Ontario, Canada using multinomial logistic regression and a case-case study approach

**DOI:** 10.1186/1471-2458-12-866

**Published:** 2012-10-12

**Authors:** Csaba Varga, Dean Middleton, Ryan Walton, Rachel Savage, Mary-Kathryn Tighe, Vanessa Allen, Rafiq Ahmed, Laura Rosella

**Affiliations:** 1 Department of Population Medicine, University of Guelph, Guelph, Ontario, N1G 2W1, Canada; 2 Ontario Ministry of Agriculture, Food, and Rural Affairs, Guelph, Ontario, N1G 4Y2, Canada; 3 Public Health Ontario, 480 University Ave, Toronto, Ontario, M5G 1V2, Canada; 4 Dalla Lana School of Public Health, University of Toronto, 155 College St., Health Sciences Building, 6th Floor, Toronto, Ontario, M5T 3M7, Canada; 5 National Microbiology Laboratory, Public Health Agency of Canada, Winnipeg, Manitoba, Canada

## Abstract

**Background:**

Identifying risk factors for *Salmonella* Enteritidis (SE) infections in Ontario will assist public health authorities to design effective control and prevention programs to reduce the burden of SE infections. Our research objective was to identify risk factors for acquiring SE infections with various phage types (PT) in Ontario, Canada. We hypothesized that certain PTs (e.g., PT8 and PT13a) have specific risk factors for infection.

**Methods:**

Our study included endemic SE cases with various PTs whose isolates were submitted to the Public Health Laboratory-Toronto from January 20th to August 12th, 2011. Cases were interviewed using a standardized questionnaire that included questions pertaining to demographics, travel history, clinical symptoms, contact with animals, and food exposures. A multinomial logistic regression method using the Generalized Linear Latent and Mixed Model procedure and a case-case study design were used to identify risk factors for acquiring SE infections with various PTs in Ontario, Canada. In the multinomial logistic regression model, the outcome variable had three categories representing human infections caused by SE PT8, PT13a, and all other SE PTs (i.e., non-PT8/non-PT13a) as a referent category to which the other two categories were compared.

**Results:**

In the multivariable model, SE PT8 was positively associated with contact with dogs (OR=2.17, 95% CI 1.01-4.68) and negatively associated with pepper consumption (OR=0.35, 95% CI 0.13-0.94), after adjusting for age categories and gender, and using exposure periods and health regions as random effects to account for clustering.

**Conclusions:**

Our study findings offer interesting hypotheses about the role of phage type-specific risk factors. Multinomial logistic regression analysis and the case-case study approach are novel methodologies to evaluate associations among SE infections with different PTs and various risk factors.

## Background

In Canada, *Salmonella* are the second most frequently reported enteric bacteria [1] and the major foodborne bacteria causing hospitalization and death [2]. In Ontario, it is estimated that for every reported *Salmonella* case, 13 to 37 cases go unreported [3]. In Ontario and Canada, integrated disease surveillance systems reported an increasing trend of *Salmonella* enterica serovar Enteritidis (SE) infections in humans showing a threefold increase between 2003 and 2009 [4]. Consequently, SE became the top nontyphoidal *Salmonella* serotype. The SE phage types (PT) influencing this increase were PT13, 8 and 13a. These PTs exhibited a seasonal, summer increase and were mostly associated with domestically acquired infections. Conversely, the number of infections with other PTs (4, 1, 6a) increased during the winter months and it was demonstrated that they were largely associated with international travel [5].

Researchers and public health professionals have traditionally used case–control study designs to investigate potential risk factors for enteric infections. One limitation of this study design is the requirement to select healthy controls, which is methodologically challenging and can increase the cost and length of the study. In addition, comparing controls selected from the general population to cases selected from a surveillance system may introduce selection and recall bias into the study [6]. Cases reported in the same surveillance system tend to be more alike due to the relatively greater reporting of certain groups (e.g., older and younger persons, people from areas with greater access to health care) and therefore are not representative of the true at risk population [7]. An increasing number of studies are using the case-case analytical approach where cases and ‘controls’ are selected from the same surveillance system. In this type of study, controls only differ from cases by their serotype (i.e., SE compared to S. typhimurium) or PT (i.e., SE PT8 compared to SE PT13). This strategy minimizes the impact of selection bias. Furthermore, the case status is laboratory-confirmed thus eliminating the possibility of undetected illnesses in controls.

Recent case-case studies of various enteric pathogens compared the risk factors for: *Salmonella* outbreak cases with other *Salmonella* cases [6], salmonellosis cases occurring during the summer with all of the other salmonellosis cases [8], campylobacteriosis cases with other enteric infections [9], *Campylobacter* coli cases with *C*. jejuni cases [10], SE cases with other *Salmonella* serotypes [11], and SE PT4 infections with cases infected by other foodborne pathogens [12].

A case–control study was undertaken in 2010–2011 to understand the reasons for the increase in the number of SE infections in Ontario. Our study uses only the case information of the domestically-acquired cases in order to identify PT - specific risk factors using multinomial logistic regression and a case-case study method. Controls were represented by non-PT8/non-PT13a infections to which SE PT8 and PT13a infections were compared. We hypothesized that risk factors for infection vary by PT. Moreover, our research study discusses the advantages and disadvantages of the case-case study using the multinomial logistic regression technique in identifying PT - specific risk factors.

## Methods

### Study background, design and laboratory methods

**Figure 1:**
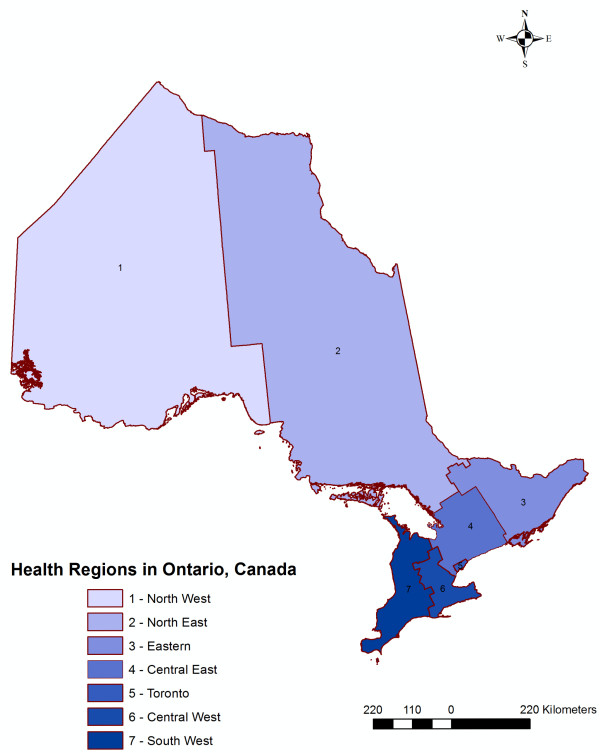
Ontario, by Health Region, 2011.

Ontario is the largest Canadian province. In 2011, there were an estimated 13.2 million people, consisting of 38.7% of the total Canadian population. All of Ontario’s residents are eligible for provincially funded health coverage. In Ontario, all clinical isolates of *Salmonella* identified by hospital, private and regional public health laboratories are sent to the Public Health Laboratory-Toronto for confirmation and serotyping. SE are confirmed based on the serological confirmation of compatible somatic and flagellar antigens (Kauffmann-White classification) [13]. All isolates serotyped as SE are forwarded to the National Microbiology Laboratory in Winnipeg, Manitoba where phage typing is performed using methods described by Ward and colleagues [14]. The serotyping and phage typing results are collected at Public Health Laboratory-Toronto and shared with epidemiologists in an ongoing line list format for surveillance purposes. In Ontario, there are 36 public health units, which administer health promotion and disease prevention programs. The 36 health units are grouped into seven Health Regions and we used these Health Regions for the purposes of our analysis (Figure 1). Salmonellosis is a Reportable Disease under provincial legislation and all infections confirmed by hospital, private, and public health laboratories must be reported to the local public health units for follow-up.

Our study used all of the exposure information pertaining to food consumption and animal contact derived from a standardized questionnaire from a case–control study (Middleton D *et al.*, unpublished results) that aimed to identify risk factors for endemic SE infections. Our study included SE isolates that were received at the Public Health Laboratory-Toronto between January 20th and August 12^th^, 2011. Cases were excluded prior to being interviewed who: resided outside of Ontario, or had SE isolated from a clinical specimen other than stool. Cases were lost to follow-up who: did not have a telephone number available, could not be reached following five attempts, or died. Refusals were defined as those cases that declined to be interviewed. Interviewed cases were excluded from this analysis who: resided on a First Nations reserve, were asymptomatic, could not recall their symptom onset date, had testing performed more than two months following symptom onset, were a secondary case (e.g., they lived with another person who had similar symptoms in the week prior to their symptom onset), were part of a recognized cluster or outbreak (not including the index case), had travelled outside of Canada and the United States of America (U.S.) within the 3 days prior to symptom onset, or could not speak English. Further, only the first specimen was included for cases that had repeated specimens purposefully performed.

Case interviews were performed by telephone using a standardized questionnaire that underwent several iterations during the hypothesis-generating stage of the study. The questionnaire included information on demographics, travel history, clinical symptoms, contact with animals, and food exposures. Food exposures included eggs (cooked versus undercooked or raw), chicken (including processed chicken), cheese, peanut butter, and select raw fruits and vegetables. Vegetables included any type of raw or uncooked pepper, carrot, onion, lettuce, spinach, and tomato. Inclusion of exposure history for the case–control study questionnaire was based on current knowledge of risk factors for SE infections and on the results of the hypothesis generating study. We asked cases about food exposures and animal contact during the three days prior to their symptom onset. Parents or guardians responded on behalf of children less than 16 years of age. All respondents gave informed verbal consent prior to beginning the interview. Under the *Ontario Health Protection and Promotion Act*[15], ethics approval was not required to interview cases because contacting the cases was considered to be part of a provincial public health investigation.

### Data management and statistical analysis

All exposure history for SE infections with different PTs pertaining to food consumption and animal contact obtained from the case-control study were entered into a spreadsheet program (Microsoft Excel 2000, Microsoft Corporation, Redmond, Washington, U.S.), were reviewed for missing values, proper coding, and distribution of values, and then imported into STATA software (Intercooled Stata 10.0; Stata Corporation, College Station, Texas). Dichotomous (yes/no) risk factor variables were created representing the food consumption history and animal contact of SE infections with various PTs (Table 1). Variables with high numbers of missing values (5% missing), low variability (less than 5% variability) or unclear answers were excluded from the analysis [16]. We considered age group, exposure period, health region, and gender to be confounders *a priori* and we therefore included them in the final models. The variable exposure period was based on the cases’ disease onset and contained three categories with an approximately equal number of cases in each category. The variable age was divided into four categories: cases aged between 0 and 9, 10 and 19, 20 and 49, and cases 50 years of age and greater. The variable health region was based on the cases’ residence and included seven Health Regions (Central-West, Central-East, Eastern Ontario, North-West, North-East, South-West and Toronto) (Figure 1).

**Table 1:** Salmonella Enteritidis, by phage type, Ontario, Canada

***Phage Type***	**N **^***a) ***^**(%)**
PT1	12 (6.0)
PT1b	1 (0.5)
PT3	1 (0.5)
PT3a	1 (0.5)
PT4	3 (1.5)
PT5b	4 (2.0)
PT6a	3 (1.5)
PT8	73 (36.7)
PT13	20 (10.1)
PT13a	50 (25.1)
PT14c	1 (0.5)
PT21c	10 (5.0)
PT22	1 (0.5)
PT34	1 (0.5)
PT41	1 (0.5)
PT51	5 (2.51)
PT atypical	9 (4.5)
PT untypable	3 (1.5)
**Total**	199 (100)

^*a)*^ N- Number of phage types.

Multinomial logistic regression analyses were utilized to analyze associations between risk factor variables and illnesses caused by different SE PTs. The outcome variable had three categories representing human infections caused by SE PT8, PT13a and non-PT8/non-PT13a. The non-PT8/non-PT13a category was considered as the base category to which the other two categories were compared.

The model building approach included three steps. First, all risk factor variables were individually regressed on the dependent variable using a univariable regression approach. Variables for which Wald’s p < 0.10 were considered for multivariable analysis. Pair-wise correlation coefficients among all unconditionally significant risk factor variables (p < 0.10) were examined. If the variables were highly correlated (rho > 0.80), to prevent collinearity the variable with the smallest p-value was considered for additional testing. Second, all unconditionally significant variables (p < 0.10) were placed in a multivariable regression model and a manual backward elimination process was employed. Third, a random intercept logistic regression model with three levels was constructed using the Generalized Linear Latent and Mixed Model (GLLAMM) procedure [17]. The hierarchical structure of the model included three levels; risk factor variables for SE PT infections at the lowest level, exposure periods at the second level, and health regions at the highest level. The model presumed that risk factor variables were conditionally independent at the lowest level of the model. The higher level exposure periods and health regions were used as random effects in order to account for clustering (absence of independence of risk factor variables and SE PT infections within exposure periods and health regions). Associations were analyzed at the individual SE PT infections level using the significant risk factor variables from the second step as well as age and gender. We measured the total unexplained variance components residing at each level of the model (i.e., SE PT infections, exposure periods, and health regions) by assuming that level 1 variance on the logit scale was π ^2^ / 3 = 3.29, π = 3.1416 [18].

## Results

### Descriptive statistics

**Figure 2:**
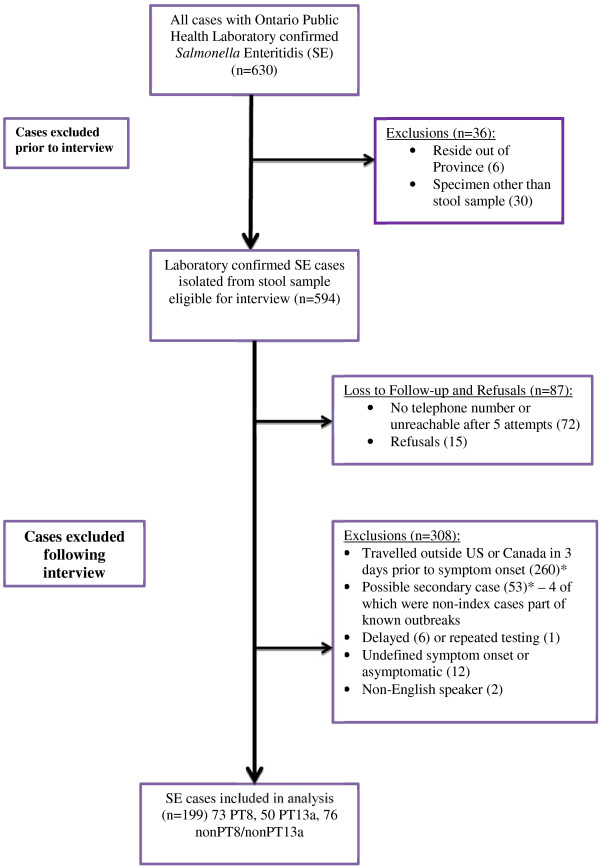
***Salmonella *Enteritidis case flow chart, Ontario, Canada, 2011.** * Indicates that 26 cases reported travel outside of North America and were also considered a potential secondary case.

**Figure 3:**
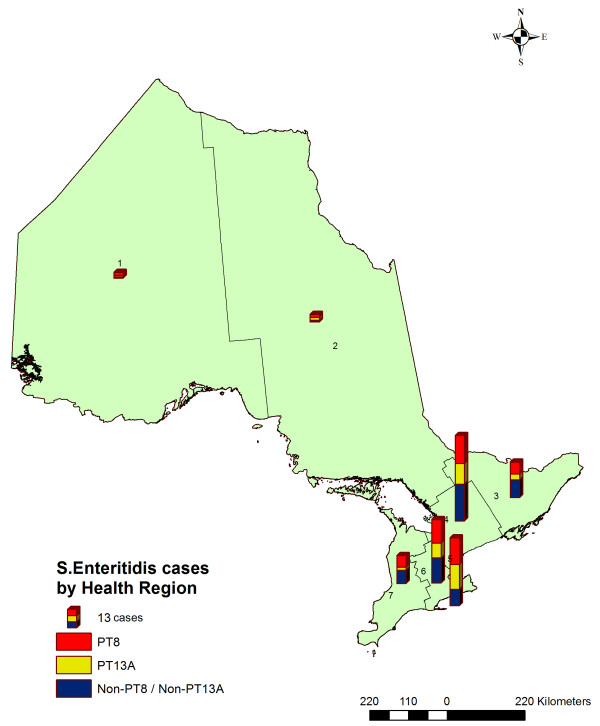
***Salmonella *Enteritidis infections, by health region and phage type, Ontario, Canada, 2011 (n = 199).**

A flow chart of case recruitment is shown in Figure 2. A total of 630 laboratory confirmed cases of SE were detected during our study. Thirty-six cases were excluded prior to interview leaving 594 cases eligible to be interviewed. A total of 87 cases were lost to follow-up or refused leaving 507 eligible cases. According to our post-interview exclusion criteria, 308 cases were excluded, leaving 199 SE cases for inclusion in our analysis. The 199 cases consisted of 73 PT8 (36.7%), 50 PT13a (25.1%), and 76 non-PT8/non-PT13a (38.1%) (Table 1). The distributions of SE infections with different PTs across Ontario’s health regions are shown in Figure 3. Visually inspecting the map it appears that the major PTs are evenly distributed across Ontario.

Symptom information was reported for 198 of the SE cases. Of these, diarrhea, abdominal cramps and fever were the most frequently reported symptoms (Table 2). The cases’ symptom onsets ranged between January 2^nd^ and August 1^st^, 2011.

**Table 2:** ***Salmonella *Enteritidis, by phage type and clinical features, Ontario, Canada (n = 199)**

***Clinical information***		***Phage type data***	
		***[Yes/N***^***a***^***(%)]***	
	**PT8**	**PT13a**	**Non-PT8/ non-PT13a**
Diarrhea	72/73 (99)	49/50 (98)	74/75 (99)
Abdominal cramps	71/73 (97)	39/50 (78)	69/75 (92)
Fever	55/68 (81)	39/48 (81)	55/70 (79)
Nausea	37/72 (51)	25/48 (52)	37/71 (52)
Vomiting	32/73 (44)	19/50 (38)	31/75 (41)
Emergency room visit	34/71 (48)	19/47 (40)	39/75 (52)
Hospitalization	11/35 (31)	7/20 (35)	14/39 (36)

^*a)*^ N-Total number of phage types.

### Multinomial logistic regression

Unconditional associations (P ≤ 0.10) were detected between any dog contact and SE PT8 infection (OR=1.90, 95% CI 0.96-3.76), any chicken consumption and SE PT13a infection(OR=0.35, 95% CI 0.13-0.99), frozen chicken consumption and SE PT8 infection (OR=4.60, 95% CI 0.87-24.22) and PT13a infection (OR=5.75, 95% CI 0.95-34.79), pepper consumption (any type of raw or uncooked pepper) and SE PT8 infection (OR=0.41, 95% CI 0.16-1.02), and tomato consumption and SE PT8 infection (OR= 0.52, 95% CI 0.26-1.04) when compared to infections caused by the non-PT8/non-PT13a group (Table 3). Unconditionally significant variables were not highly correlated; therefore all variables were included in the multivariable model.

**Table 3:** **Unconditional associations among risk factors and human *Salmonella *Enteritidis infections by phage type, Ontario, Canada (n = 199)**

***Exposure***	***Outcome [Yes/No] ***^***†***^	***OR ***^***a)***^***(95% CI ***^***b***^***) ***^***†***^
Any dog contact	Non-PT8/non-PT13a [22/48)]	-
	PT8 [34/39]	1.90 (0.96-3.76) *
	PT13a [15/34]	0.96 (0.44-2.12)
Any cat contact	Non-PT8/non-PT13a [18/54]	-
	PT8 [21/52]	1.21 (0.58-2.53)
	PT13a [9/40]	0.68 (0.27-1.66)
Any egg consumption	Non-PT8/non-PT13a [34/35]	-
	PT8 [38/28]	1.40 (0.71-2.75)
	PT13a [26/20]	1.34 (0.63-2.83)
Runny egg consumption	Non-PT8/non-PT13a [11/20]	-
	PT8[11/21]	0.95 (0.34-2.68)
	PT13a [7/16]	0.80 (0.25-2.52)
Undercooked egg consumption	Non-PT8/non-PT13a [11/19]	-
	PT8 [12/19]	1.09 (0.39-3.07)
	PT13a [8/15]	0.92 (0.30-2.87)
Egg consumption away from home	Non-PT8/non-PT13a [10/24]	-
	PT8 [13/24]	1.30 (0.48-3.53)
	PT13a [9/17]	1.27 (0.43-3.79)
Any chicken consumption	Non-PT8/non-PT13a [61/7]	-
	PT8 [55/14]	0.45 (0.17-1.20)
	PT13a [34/11]	0.35 (0.13-0.99) *
Processed chicken consumption	Non-PT8/non-PT13a [7/20]	-
	PT8 [4/22]	0.52 (0.13-2.04)
	PT13a [4/11]	1.04 (0.25-4.35)
Fresh chicken consumption	Non-PT8/non-PT13a [21/6]	-
	PT8 [19/6]	0.90 (0.25-3.29)
	PT13a [12/3]	1.14 (0.24-5.42)
Frozen chicken consumption	Non-PT8/non-PT13a [2/23]	-
	PT8 [8/20]	4.60 (0.87-24.22) *
	PT13a [5/10]	5.75 (0.95-34.79) *
Fast food chicken consumption	Non-PT8/non-PT13a [10/48]	-
	PT8 [12/39]	1.48 (0.58-3.78)
	PT13a [8/24]	1.60 (0.60-4.58)
Chicken consumption away from home	Non-PT8/non-PT13a [37/23]	^-^
	PT8 [32/22]	0.90 (0.43-1.92)
	PT13a [21/13]	1.00 (0.42-2.39)
Cheese consumption	Non-PT8/non-PT13a [50/16]	-
	PT8 [47/20]	0.75 (0.35-1.62)
	PT13a [32/12]	0.85 (0.36-2.04)
Peanut butter consumption	Non-PT8/non-PT13a [16/51]	-
	PT8 [23/46]	1.59 (0.75-3.38)
	PT13a [8/36]	0.71 (0.27-1.83)
Carrot consumption	Non-PT8/non-PT13a [25/44]	-
	PT8 [21/45]	0.82 (0.40-1.68)
	PT13a [16/31]	0.91 (0.42-1.98)
Pepper consumption	Non-PT8/non-PT13a [17/52]	-
	PT8 [8/60]	0.41 (0.16-1.02) *
	PT13a [9/36]	0.76 (0.31-1.91)
Onion consumption	Non-PT8/non-PT13a [13/57]	-
	PT8 [15/53]	1.24 (0.54-2.85)
	PT13a [9/36]	1.20 (0.43-2.83)
Lettuce consumption	Non-PT8/non-PT13a [40/26]	-
	PT8 [41/26]	1.03 (0.51-2.06)
	PT13a [26/18]	0.94(0.43-2.04)
Spinach consumption	Non-PT8/non-PT13a [6/63]	-
	PT8 [10/60]	1.75 (0.60-5.11)
	PT13a [4/42]	1.00 (0.27-3.76)
Tomato consumption	Non-PT8/non-PT13a [37/32]	-
	PT8 [24/40]	0.52 (0.26-1.04) *
	PT13a [23/22]	0.90 (0.43-1.92)
Strawberry consumption	Non-PT8/non-PT13a [27/41]	-
	PT8 [22/44]	0.76 (0.37-1.54)
	PT13a [10/31]	0.49 (0.21-1.16)

^***†***^ Univariable multinomial logistic regression model, comparing PT8 and PT13a to non-PT8/non-PT13a. ^a) ^*OR* - Odds Ratio. ^b) ^*CI* – Confidence Interval of the OR. * Variables with P ≤ 0.1 were used for the multivariable analysis.

In the multivariable model, SE PT8 infection was positively associated with contact with dogs (OR=2.17, 95% CI 1.01-4.68) and negatively associated with pepper consumption (OR=0.35, 95% CI 0.13-0.94), when compared to the non-PT8/non-PT13a group, after adjusting for age categories and gender, and using exposure periods and health regions as random effects. No significant associations were detected among risk factors and SE PT13a infection when compared to the non-PT8/non-PT13a group (Table 4).

**Table 4:** **Risk factors for *Salmonella *Enteritidis by phage type, Ontario, Canada (n=199) **^***†***^

***Outcome ***^***a)***^	***Exposure***	***OR ***^***b)***^***(95% CI ***^***c)***^***)***	P ^*^
PT8	Any dog contact	2.17 (1.01-4.68)	0.047
	Pepper consumption	0.35 (0.13-0.94)	0.038
PT13a	Any dog contact	0.96 (0.40-2.31)	0.928
	Pepper consumption	0.61 (0.23-1.62)	0.321

^***†***^ Final multivariable GLLAMM model adjusted for age groups and gender. Health regions and exposure periods used as random effects. ^a)^ PT8, and PT13a, compared to the non-PT8/non-PT13a group. ^b) ^*OR* - Odds Ratio. ^c) ^*CI* – Confidence Interval of the OR. ^*^ Statistically significant at P ≤ 0.05.

The total amount of unexplained variation obtained from the multilevel logistic regression GLLAMM model was 3.69, from which 3.29 (89.2%) resided at the individual SE PT risk factor level, 0.38 (10.3 %) at the exposure period level, and 0.02 (0.5%) at the health region level.

## Discussion

Multinomial regression methodology is rarely used in foodborne disease investigations, and to our knowledge it was used only once previously in a U.S. case–control study where researchers compared various SE PT infections to healthy controls [19]. To the best of our knowledge, our study is the first study worldwide to use multinomial logistic regression and the case-case study approach to evaluate associations among SE infections with different PTs and various risk factors. Moreover, our study used a three level random intercept logistic regression model with the GLLAMM procedure. The advantage of the GLLAMM model is that it adjusts for the variation in risk factors for SE PT infections across exposure periods and health regions and it allows for the measurement of unexplained variation in SE PT infection risk factors at these levels. From the total unexplained variation, a moderate amount (10.3%) resided at the exposure period level indicating seasonal clustering of SE PT infection risk factors. Only a small proportion (0.5%) of the total variation resided at the health region level indicating no spatial clustering of cases. This may be explained by the possibility that risk factor variables were evenly distributed across the study area.

In the multivariable model, statistically significant associations were demonstrated between contact with dogs during the three days before disease onset and infection with SE PT8 when compared to the non-PT8/non-PT13a groups, while accounting for age groups and gender, and using exposure periods and health regions as random effects to account for clustering. Several studies have previously demonstrated associations between human salmonellosis and animal contact. Examples include contact with reptiles [19-22], cats [22], and pet birds [19]. Moreover, it is well documented that owning a dog can be a risk factor for acquiring salmonellosis. Behravesh *et al.* demonstrated that feeding a dog in the household with dry food was a risk factor for *Salmonella* Schwarzengrund infections [23]. Several studies showed that feeding raw foods to dogs increased their likelihood of *Salmonella* shedding and consequently the risk of infecting the environment and possibly humans [24-28]. Findings from our study are consistent with the studies above. It should be noted, however, that these studies used different analytical methods and *Salmonella* Enteritidis was not among the identified *Salmonella* serotypes. More research is required to understand SE disease transmission from dogs to humans, and more specifically, to confirm whether there is a relation between SE PT8 infection and exposure to dogs in order to assist public health authorities with designing SE PT - specific prevention and control programs.

Consumption of peppers was significantly associated as having a protective effect for SE PT8 cases when compared to the non-PT8/non-PT13a cases. This result could also indicate that consuming peppers is a risk factor for SE infections with the non-PT8/non-PT13a cases. Historically foodborne disease outbreaks including outbreaks caused by various *Salmonella* serotypes have been most frequently caused by consumption of foods of animal origin, but more recently an increased number of foodborne outbreaks have been associated with consumption of fresh fruits and vegetables [29-31]. In a U.S. multistate outbreak investigation, public health authorities identified a link between raw jalapeño pepper consumption and *Salmonella* Saintpaul infections [32].

In the univariable unconditional model, the odds ratios for frozen chicken consumption and PT8 and PT13a infections compared to non PT8/non PT13a infections were relatively high (4.60 and 5.75, respectively) however not statistically significant. We believe the lack of statistical significance is likely due to a lack of power in the study or that chicken consumption was spread equally among the PT comparison groups. Our finding is not surprising knowing that PT13a and PT8 are among the most frequently SE PTs identified from retail chicken through the Canadian integrated surveillance systems [4]. Associations between chicken consumption and SE PT8 and SE PT13a infections have also been demonstrated in studies from the U.S. [11,19,33].

Our study only identified two statistically significant risk factors at the multivariable level and these risk factors were both associated with SE infections caused by PT8. The inability to identify other statistically significant risk factors could be explained by these risk factors being distributed evenly across the PT comparison groups (i.e., PT8, PT13a, and non-PT8/non-PT13a). If this was the case, the case-case study design would not be able to demonstrate differences among the given risk factors and the various PT comparison groups. It is also possible that the two statistically significant findings occurred by chance alone, or there were confounder variables that we did not include in our analysis.

There are several advantages to using the multinomial case-case technique. One clear advantage is that for one particular pathogen (e.g., SE), the laboratory sub-type of interest (e.g., PT8) can be compared to other sub-types (e.g., PT 13a, non PT8/non PT13a) within the same model. Consequently, the model can analyze more than two outcomes simultaneously. A second advantage is that the methodology uses case-case comparisons that allow inferences to be made using only case exposure data. Thus, the effort required to collect control data is not required and, therefore, less time and fewer resources are required to complete the study. This could be particularly applicable in food borne outbreak investigations. If a food item is causing an above expected amount of illnesses of a frequently reported pathogen subtype (i.e., a PT or a pulsed-field gel electrophoresis pattern), the subtype could be compared to the “other” subtypes in a relatively efficient manner, assuming the food item is causing illness in expected amounts in the “other” subtypes and there is an adequate number of cases in the other subtype groupings to provide sufficient power. This method has been previously used efficiently in the U.S. during multistate listeriosis outbreak investigations [34,35].

A third advantage of case-case methodology is that the study design reduces the selection and recall bias usually experienced in case–control studies.

There are several disadvantages in using a case-case multinomial methodology. One disadvantage is that the pathogen subtyping comparison groups (e.g., SE PT8 vs. the non-PT8s) may have similar risk factors [36]. If this is the case, the methodology would fail to identify the risk factor as associated with either PT group. As noted above, in our study the risk factors for SE may have been evenly distributed among the three PT groups and thus significant differences could not be detected. It is important to note that the case-case approach tested the difference between three SE PT groups as opposed to testing the difference between cases infected with SE and those not infected, which is assessed in a traditional case–control design. Therefore, different interpretations may occur for the same risk factor depending on which study design is being employed. Case-case analysis may only demonstrate that infections with certain PTs are associated with certain risk factors compared with other risk factors. Thus, one must carefully interpret the results of the two different study designs. If the case-case methodology is used more frequently, a greater understanding of the advantages and disadvantages in regard to interpreting the outcome of each methodology will be required.

## Conclusion

In conclusion, we offer insight into the advantages and disadvantages of multinomial case-case analysis applied to sporadic cases of SE. Compared to a traditional case–control study, a case-case analysis has the advantage of not requiring the time and resources to obtain healthy controls. The case-case methodology may be more effectively used in outbreaks, rather than for sporadic cases, when there is potentialy one food item causing the majority of cases of a certain subtype (e.g., SE PT8). Further investigations are needed to confirm our study findings and assess their usefulness in regard to public health control measures for SE.

## Abbreviations

GLLAMM: Generalized linear latent and mixed model; PT: Phage type; SE: *Salmonella* enteritidis; U.S.: United States.

## Competing interests

The authors declare that they have no competing interests.

## Authors' contributions

CV, RW, and DM conceived of the study. CV, RW, LR and DM developed the study design and methodology. MKT and RS were involved with interviewing cases. CV was involved with data management and analysis. RW, RS, LR, and DM provided advice on the data analysis. VA and RA were involved in laboratory analysis. CV, DM, MKT, RS, RW,and LR were involved with drafting the manuscript. All authors read and approved the final manuscript.

## Pre-publication history

The pre-publication history for this paper can be accessed here:

http://www.biomedcentral.com/1471-2458/12/866/prepub
